# Validity and Reliability of the Index of Self-Regulation Scale for Physical Activity in Older Korean Americans

**DOI:** 10.1155/2011/329534

**Published:** 2011-05-30

**Authors:** Hye-A Yeom, Julie Fleury

**Affiliations:** ^1^The Catholic University of Korea College of Nursing, Seoul 137-701, Republic of Korea; ^2^Arizona State University College of Nursing and Health Innovation, AZ 85004, USA

## Abstract

The Korean version of the index of self-regulation (KISR) is a nine-item scale designed to measure individuals' level of self-regulation for physical activity. The purpose of this study was to test the psychometric properties of the KISR, including reliability and validity, in a group of older Korean Americans. The KISR was administered to a sample of older Korean Americans at a baseline interview (Time 1) and 12 week followup (Time 2). The internal consistency of the KISR was high at both time points, with Cronbach's alphas of .94 and .95, respectively. The test-retest reliability was moderate-to-high at .68. There was evidence of construct validity of the KISR based on its moderate to high significant correlations with theoretically relevant variables, including motivational appraisal and self-efficacy for physical activity. A principal axis factoring with an oblique rotation resulted in two factors, explaining 89% of the variance. The KISR is a reliable and valid measure to assess the level of self-regulation for physical activity behavior in older Korean Americans.

## 1. Introduction

Regular physical activity is defined as a planned, structured, energy-consuming activity performed on a repeated basis, with specific health goals including improved physical functioning and/or fitness [1]. Current clinical guidelines suggest that adults engage in 30 minutes of moderate intensity physical activity (PA) on all or most days of the week for health promotion and maintenance [2]. Despite the health benefits of regular physical activity in reducing the risk of cardiovascular disease (CVD), diabetes, obesity, and other chronic conditions, approximately 50% of older adults do not participate in regular PA and have no intention to initiate regular physical activity [3]. 

Korean Americans are one of the most rapidly growing immigrant populations in the United States. Among older Korean Americans, high blood pressure has been identified as a leading risk factor for cardiovascular disease (CVD), followed by high blood cholesterol and overweight [4]. When compared to National Health and Nutrition Examination Survey (NHANES III) data, the prevalence of high blood pressure among Korean American elders (71.2%) was higher than in whites and Hispanics, but it was similar to that for African Americans. Data from the California Health Interview Survey [5] indicate that Asian Americans were much less likely to meet recommended levels of leisure time physical activity and had lower estimated weekly energy expenditure than US-born non-Asians.

Self-regulation, a psychological concept that has been broadly studied across disciplines, including behavioral medicine and social sciences, is recognized as important to fostering personal control, goal-directed behaviors through selective processing of information, behavioral monitoring, judging individual performance, and self-evaluation [6–8]. Self-regulation has been used to describe and predict adherence to cardiovascular risk-reducing behaviors, including regular physical activity [7, 9, 10]. Self-regulatory mechanisms are key to understanding volitional aspects of behavior change in that they reflect the ways in which people attempt to behave in accordance with personal goals, particularly when goals conflict or lead to differential rewards over time. Brawley and colleagues [11] emphasize self-regulation skills as essential to promoting physical activity maintenance in older adults. In a convenience sample of older adults, Umstattd and colleagues [12] found that self-regulatory strategies were associated with all forms of current physical activity participation, including moderate-to-vigorous activity. 

The index of self-regulation (ISR), an English-language questionnaire developed by Fleury [13], is a nine-item scale designed to measure level of self-regulation for health behavior change. The initial step in instrument development consisted of an exploratory study to identify the psychological and social processes used to initiate and sustain health behavior over time [14]. Qualitative data explicated the meaning of self-regulation as a concept central to the maintenance of behavioral change and provided a conceptual basis for instrument development, including the subconcepts of reconditioning, stimulus control, and self-monitoring. Reconditioning reflects attempts to narrow the range of stimuli associated with risk-producing behavior; stimulus control indicates attempts to strengthen maintenance of behavioral change through focusing on positive aspects of risk modification, and behavioral monitoring refers to the assessment of adherence to self-determined criteria for goal achievement. Psychometric properties of the ISR have been supported in health promotion studies with ethnically diverse populations [13, 15, 16]. Subscale alpha reliability has ranged from  .72 to  .86. Construct validity was examined though path analysis correlating ISR subscales with theoretically related constructs of motivation appraisal and the performance of physical activity [13, 17]. Efforts to develop and evaluate the psychometric properties of the ISR have been successful and suggest that the ISR is a promising tool for measuring self-regulation for maintenance of behavioral change. 

Although the concept of self-regulation holds promise for fostering long-term health behavior change, reliable and valid measures of self-regulatory skills related to the maintenance of physical activity in older Korean Americans have yet to be developed and tested. Therefore, the purpose of this study was to test the psychometric properties of the Korean version of the index of self-regulation (KISR), including reliability and validity, in a group of older Korean Americans residing in the U.S.

## 2. Theoretical Perspective

The conceptual meaning of self-regulation was examined within the context of the wellness motivation theory (WMT) [18, 19], which includes an explication of the essential features of the concept and its relationship to social contextual influence, behavioral change process, and behavioral outcome variables within the theory. The WMT includes an analysis of how people generate goals, imagine opportunities for action, and create and execute strategies for health-relevant behavioral change. Within the WMT, behavioral change is conceptualized as a process of forming intentions and engaging in goal-directed behavior that activates and guides positive health patterns. The WMT consists of three dimensions: social contextual influences (social support and environmental resources), behavioral change process variables (self-knowledge, motivational appraisal, self-regulation), and action (physical activity). These dimensions acknowledge the interaction of the person, the environment, and health behaviors and reflect a motivational process that includes the identification of personal values, strengths, resources, and strategies that result in purposeful action and subsequent self-regulation processes.

Self-regulation is a concept integral to the WMT, which reflects the cognitive, affective, and behavioral strategies for behavioral change congruent with valued goals. The pursuit and attainment of self-generated goals and the maintenance of self-determined standards for behavior are critical sources of motivation that involve the internal regulation of behavior. Self-regulation includes the evaluation of response to social contextual influences, and regulation and evaluation of personal efforts at self-management. Within the WMT, self-regulation, self-efficacy, and motivation appraisal reflect behavioral change process variables which are theoretically positively related. For testing the construct validity of the KISR, these variables were tested for their relationship with self-regulation. Because these are conceptually interrelated, positive relationships are expected to support construct validity of the KISR.

## 3. Method

### 3.1. Design and Setting

To assess the reliability and validity of the KISR, testing of the scale was conducted in a sample of older Korean Americans. Participants were a convenience sample recruited from Korean ethnic churches in Arizona. Participants completed the KISR, measures of motivational appraisal and self-efficacy, and provided relevant demographic information. The KISR was administered at study baseline (Time 1) and 12 weeks later (Time 2). The data collected at Time 1 were to examine item characteristics, internal consistency, and construct validity of the KISR; data collected at Time 2 were used to evaluate test-retest reliability. The KISR was administered via person-to-person interviews at the participant's own home or ethnic church, as preferred by the participant.

### 3.2. Sample

The study participants included a total of 68 community-dwelling older Korean Americans. Subject eligibility criteria included a senior person with Korean heritage who was (a) 60 years of age or older, (b) able to communicate in Korean or English, (c) willing to participate in the study voluntarily, and (d) at low risk for participation in moderate intensity physical activity as indicated by the physical activity readiness questionnaire (PAR-Q) [20].

### 3.3. Data Collection

Participants were invited to participate in the study through poster presentations at community churches. During an initial meeting, participants were informed of the study purpose and protocol. Those who met eligibility criteria and who agreed to participate reviewed and signed the study consent form. Following informed consent, participants were administered a questionnaire packet containing the KISR, measures of self-efficacy and motivational appraisal, and a demographic data form for completion. The study site coordinator reviewed the measures for completeness and answered any questions the participant might have. This protocol was repeated in 12 weeks.

### 3.4. Measures

Measures operationalized self-regulation and theoretically relevant variables (self-efficacy and motivational appraisal) to evaluate reliability and construct validity of the KISR.

#### 3.4.1. Self-Regulation

Self-regulation was measured using the KISR, which reflects individual strategies for regulating behavioral change to engage in regular physical activity. Translation and cultural adaptation of the KISR was conducted, including forward translation by a native Korean speaker with experience in translation, reconciliation, back translation by native Korean speakers, back translation review, and cognitive debriefing [21]. These steps supported the quality of translation, level of comprehensibility, content relevance, and the conceptual equivalence of the translation. The KISR consists of 9 items on a Likert scale ranging from 1 (strongly disagree) to 6 (strongly agree). Completion of the KISR is expected to take 5 to 10 minutes. A higher sum score indicates a higher level of self-regulation for physical activity.

#### 3.4.2. Self-Efficacy

Self-efficacy for physical activity was measured using exercise for self-efficacy (ESE) scale developed by Resnick and Jenkins [22]. The ESE was designed to measure a person's internal level of self-efficacy to engage in regular physical activity and has demonstrated reliability and validity in previous research on physical activity in minority older adults [23]. The maximum score for the ESE is 10; a higher score indicates a higher level of self-efficacy for physical activity.

#### 3.4.3. Motivational Appraisal

Motivational appraisal was measured using the index of readiness (IR) [24]. The IR reflects the level of readiness to initiate and maintain regular physical activity. The IR includes nine items on a Likert scale ranging from 1 (strongly disagree) to 6 (strongly agree). The IR was scored by summing the numerical ratings for each response. A higher score indicates a higher level of readiness for regular physical activity. Total scale internal consistency estimates have been established at  .80 [18].

### 3.5. Data Analysis

An analysis of missing values of variables used for construct validity was conducted. Demographic information including age, income, education, marital status, and number of health problems was described using descriptive statistics. Item and scale characteristics were evaluated using descriptive statistics. The reliability of the KISR was tested through internal consistency and test-retest consistency. Internal consistency was measured using Cronbach's alpha. Evidence of discriminate value of each item was assessed based on a coefficient between >.30 and <.70 on the examination of item-to-item correlations [25]. For the examination of test-retest reliability, the KISR was reassessed on 30 of 68 participants at 12 weeks (Time 2). This period is more than two weeks suggested by Streiner and Norman [26] and was of sufficient length to minimize memory effect. Test-retest reliability was calculated using the differences in KISR scores at Time 1 and Time 2 and the intraclass correlation coefficient (ICC). Construct validity of the KISR was evaluated through concurrent validity and factor analysis. Concurrent validity was assessed by examining the relationship between self-regulation for physical activity and conceptually relevant behavioral variables including self-efficacy and motivational appraisal for physical activity. Bivariate correlations using Pearson correlation were used to examine the relationship between these variables. Exploratory factor analysis was also conducted to identify the structure of the KISR. The data were analyzed using SPSS 12.0 version.

## 4. Results

### 4.1. Demographic Characteristics

Sixty eight older Korean adults participated in the study. The average age of the participants was 72 years, ranging from 60 to 89 years. The sample included 50 females (74%) and 18 males (26%). The majority of participants were married (60%) and were retired (93%). The mean year of residency in the U.S. was 24 years. More than half of the participants had a least a high school education (64%). Approximately 85% of the participants were taking at least one or more prescription medicine in a daily basis for the management of chronic illnesses.

### 4.2. Item Characteristics

The mean score of the KISR total scale was 22.9 at Time 1 and 21.9 at Time 2. Individual item scores ranged from 3.15 (I think of the benefits of regular physical activity), to 2.07 (I have learned new habits that help me to participate in physical activity) (Table 1). Item-to-item correlations ranged from  .39 to  .93, with a mean correlation of  .62. The average item-to-total correlation was  .78.

### 4.3. Reliability

The reliability of the KISR was tested through internal consistency and test-retest reliability. The Cronbach's alpha coefficient was  .94 at Time 1. Item-to-item correlations ranged from  .39 to  .93, with the mean correlation of  .62. Analysis of item-to-total correlations showed that item 1 (I think of the benefits of regular physical activity) had the lowest item-to-total correlation as  .62. Item 6 (I monitor myself to see if I am meeting my goals for physical activity) had the highest item-to-total correlation as  .89 (Table 1). The average item-to-total correlation was  .78. Cronbach's alpha for the KISR at Time 2 was  .95, supporting the evidence for the internal consistency of the KISR at both time points. Item-to-item correlations at Time 2 ranged from  .58 to  .91, with the mean correlation of  .82. Test-retest reliability evaluates repeatability of responses for a measure when the concept is assessed repeatedly within a certain time interval. Test-retest reliability coefficients of the KISR was  .67 (*P* = .000) between Time 1 and Time 2, showing a moderately high level of correlation between the two time points.

### 4.4. Construct Validity

Construct validity of the KISR was evaluated by examining concurrent validity and factor structure. For evaluation of concurrent validity, correlation analysis was used to examine the relationship among KISR, ESE, and the index of readiness. These variables were selected to determine the extent to which the KISR demonstrated the same direction and magnitude of correlation. Overall, concurrent validity of the KISR was supported, with significant positive correlations between self-regulation and self-efficacy for physical activity (*R* = .35, *P* = .003), as well as self-regulation and motivational appraisal (*R* = .60, *P* = .000) (Table 2). 

A principal axis factoring with an oblique rotation was conducted to validate the KISR by demonstrating its item loads on the same factor. Initial Eigenvalue <1 measuring the amount of variation in the total sample accounted for by each factor was used. Two factors with Eignvalues over 1 were extracted, explaining 89% of the variance. Factor 1 showed an Eigenvalue of 6.23, and factor 2 showed an Eigenvalue of 1.79. Factor 3 was eliminated from the factor list due to an Eigenvalue of less than 1 (.36). Factor 1 was the primary factor explaining 69% of the variance, and factor 2 explained an additional 19% of the variance. Item loadings on the two factors were evaluated based on the criteria suggested by Carmines and Zeller [27] that an item should have a factor loading of over  .45 and a difference between an item loading on the primary factor and any other factor is at least  .20. Factor 1 included four items (KISR item 1 to 4) and factor 2 included 5 items (KISR item 5 to 9).

## 5. Discussion

This study was designed to test psychometric characteristics of Korean version of the index of self-regulation (KISR) by testing its reliability and validity in a group of older Korean Americans. The reliability and validity of the KISR were evaluated in 68 community-dwelling older Korean adults. No participants reported any challenge in comprehending the meanings of the KISR items during data collection. The overall level of self-regulation for physical activity among older Korean Americans was low; with the individual item mean scores of 2.5 of 6.0, and KISR total scale means of 22.9 at Time 1 and 21.9 at Time 2. The range of individual item scores was moderate, as 2.07 to 3.15, which suggests that self-regulation for physical activity among community-dwelling older Korean Americans is moderate to low. Item scores indicate that older Korean Americans recognize the importance of regular physical activity but may experience difficulty with establishing a routine of regular physical activity, consistent with findings reporting low levels of physical activity among older Korean Americans [28, 29]. The average item-to-item correlations was moderate to high at  .62, meeting the desirable standard of >.30 or <.70 [25]. This indicates that the nine items are moderately correlated but also are heterogenic without overt similarity.

The study supports the conclusion that the KISR is a reliable instrument to measure the concept of self-regulation for physical activity in older Korean adults. The Cronbach's alpha coefficients of the KISR were high, with a mean of  .94 at Time 1 and  .95 at Time 2. These findings are consistent with those of previous studies in which  .87 and  .90 of ISR internal consistencies were reported [16, 17]. The average item-to-item correlations was fairly moderate (.62), meeting the desirable standard of >.30 or <.70 [25]. The KISR had a test-retest reliability of  .67, showing a moderate level of stability over time.

The construct validity of the KISR was examined by testing concurrent validity and factor analysis. Concurrent validity is one type of criterion validity and refers to the degree to which scores on an instrument are associated with other theoretically relevant external criterion [30]. Concurrent validity was supported by the significant positive correlation of the KISR with theoretically relevant measures of WMT concepts, self-efficacy for physical activity, and motivational appraisal. The magnitude of the correlations between the constructs was moderate to high, supporting the conceptual meaningfulness of the relationships. This finding is consistent with the findings of other researchers that self-regulation is related to behavior change variables, including self-efficacy and motivational appraisal [31–33]. 

The results of factor analysis indicated that the KISR consisted of two subdomains conceptually consistent with reconditioning, or attempts to narrow the range of stimuli associated with risk-producing behavior (Factor 1), and behavioral monitoring, or assessment of adherence to self-determined criteria for goal achievement (Factor 2). Factor 1 consists of four items, including “I think of the benefits of regular physical activity” (KISR item 1), “I remind myself of the good that I am doing by staying physically active” (KISR item 2), “I remind myself of the importance of physical activity” (KISR item 3), and “I keep track of the ways that I stay physically active” (KISR item 4). Factor 2 consists of five items, including “I watch for signs of progress as I stay physically active” (KISR item 5), “I monitor myself to see if I am meeting my goals for physical activity” (KISR item 6), “I have learned new habits that help me to participate in physical activity” (KISR item 7), “I have learned new ways to stay physically active” (KISR item 8), and “I have learned to make changes in my physical activity that I can live with” (KISR item 9). The factor structure of the KISR identified in this study was somewhat different from that of the original English version ISR reported in the previous studies [13, 15]. However, a direct comparison of the factor solutions across studies is challenging due to the different racial and age profiles of participants. Further testing of construct validity of the ISR is needed in future cross-cultural studies. Because studies using the ISR, and particularly the KISR, are limited, definitive conclusions about construct validity of the instrument are also limited. Further psychometric testing of the KISR is needed to strengthen support for construct validity. However, findings of this study support the relevance of the KISR when exploring self-regulation for physical activity behaviors in older Korean Americans. 

In conclusion, the KISR is a reliable, valid measure to assess the level of self-regulation for physical activity in older Korean Americans. The KISR is a social cognitive scale that can be used in future cross-cultural studies on physical activity behaviors of older Korean Americans.

## Figures and Tables

**Table 1:** KISR item means, standard deviations, and correlations with total (*n* = 68).

	Mean	SD	Item-Total correlation
KISR Item 1	3.15	.554	.626
KISR Item 2	3.09	.617	.678
KISR Item 3	3.00	.646	.751
KISR Item 4	2.90	.694	.666
KISR Item 5	2.19	.738	.866
KISR Item 6	2.15	.778	.894
KISR Item 7	2.07	.759	.870
KISR Item 8	2.12	.802	.864
KISR Item 9	2.09	.824	.819

**Table 2:** Correlations of KISR with ESE and index of readiness for physical activity.

		Index of readiness	Self-efficacy for exercise
Time 1	KISR	*R* = .60	*R* = .35
		*P* = .000	*P* = .003

Time 2	KISR	*R* = .55	*R* = .47
		*P* = .002	*P* = .009
